# A Deep-Sea Bacterium Senses Blue Light via a BLUF-Dependent Pathway

**DOI:** 10.1128/msystems.01279-21

**Published:** 2022-02-01

**Authors:** Yeqi Shan, Ge Liu, Ruining Cai, Rui Liu, Rikuan Zheng, Chaomin Sun

**Affiliations:** a CAS and Shandong Province Key Laboratory of Experimental Marine Biology & Center of Deep Sea Research, Institute of Oceanology, Chinese Academy of Sciences, Qingdao, China; b Laboratory for Marine Biology and Biotechnology, Qingdao National Laboratory for Marine Science and Technology, Qingdao, China; c College of Earth Science, University of Chinese Academy of Sciences, Beijing, China; d Center of Ocean Mega-Science, Chinese Academy of Sciences, Qingdao, China; University of Hawaii at Manoa

**Keywords:** deep sea, nonphotosynthetic bacterium, blue light, BLUF, light sensing

## Abstract

Light is a ubiquitous energy source and environmental signal that broadly impacts the lifestyle of a large number of photosynthetic/nonphotosynthetic microorganisms living in the euphotic layer. However, the responses of deep-sea microbes to light are largely unknown, even though blue light is proposed to be distributed in the deep ocean. Here, we successfully cultured a novel bacterial species, named *Spongiibacter nanhainus* CSC3.9, from deep-sea cold seep samples by a blue light induction approach. The growth of strain CSC3.9 was obviously promoted by the illumination of blue light. We next determined BLUF (a typical blue light photoreceptor) was the most essential factor directing light sensing of strain CSC3.9 through a combined proteomic and genetic method. The function of light sensing mediated by BLUF was further confirmed by the *in vitro*-synthesized protein. Notably, homologs of BLUF widely existed across the marine microorganisms (containing *Spongiibacter* species) derived from different environments, including cold seeps. This strongly indicates that the distribution of light utilization by the nonphototrophic bacteria living in the ocean is broad and has been substantially underestimated.

**IMPORTANCE** Extensive studies have been conducted to explore the mechanisms of light sensing and utilization by microorganisms that live in the photic zone. Strikingly, accumulated evidence shows that light is distributed in the deep biosphere. However, the existence and process of light sensing and utilization by microbes inhabiting the deep ocean have been seldom reported. In the present study, a novel bacterial strain, *Spongiibacter nanhainus* CSC3.9, was enriched and purified from a deep-sea cold seep sample through a blue light induction method. Combined with genomic, proteomic, genetic, and biochemical approaches, the mechanism of this novel strain sensing blue light through a BLUF-dependent pathway was detailed. Our study provides a good model to study the mechanisms of light sensing mediated by deep-sea nonphototrophic bacteria.

## INTRODUCTION

Light is a rich, yet variable, source of energy for organisms (1). It is therefore no surprise that phototrophic bacteria have evolved a set of sophisticated photosynthetic systems to harness light and thereby generate energy for growth (2). For a long time, most microorganisms were considered insensitive to light, with the exception of some phototrophs that use sunlight as an energy source (3). However, the view that only phototrophic microbes can sense light was challenged by the discovery of red-light sensors, bacteriophytochromes, in the nonphototrophic microorganisms Deinococcus radiodurans and Pseudomonas aeruginosa in 1999 (4). Photosensory proteins, as receptors of light, perceive and transmit the color and intensity of signals from ambient light (3). Subsequent studies have revealed that photosensors of other classes are functional in nonphototrophic bacteria, including the light-oxygen-voltage (LOV) domain protein (blue light sensing) (5), photoactive yellow protein (PYP) (blue light sensing) (6), blue light using flavin-adenine dinucleotide (FAD) (BLUF) domain proteins (7), and bacteriophytochrome (infrared light sensing) (8). BLUF was even proposed to function in a marine photosynthetic gammaproteobacterium (Congregibacter litoralis KT71^T^) (9–11). Now it is widely accepted that photosensing ubiquitously distributes in chemotrophic, nonphototrophic bacteria to perceive light in signaling pathways that regulate various cellular processes, such as pigment biosynthesis, stress response, DNA repair, host-pathogen interactions, and phototaxis (12). Phototaxis behavior is a manifestation of phototrophic microorganisms to respond to optimal illumination conditions by cell motility (13, 14). The interference of blue light in bacterial movement showed positive and negative phototaxis, which were found in photosynthetic microbes, including *Synechocystis*, *Rhodobacter*, and *Anabaena* (15, 16). On the other hand, the typical nonphototrophic bacterium Escherichia coli also showed directed migration induced by blue light (13). The phototaxis behavior in E. coli is strictly regulated by a chemotaxis-related network: CheA, as a histidine protein kinase, produces and transmits phosphorylation signals; with the aid of CheW, phosphorylation signals are transmitted to CheY, a response regulator that connects flagellum movement, thereby altering cell motor behavior (17–19).

Among different wavelengths of light, blue light ubiquitously exists on the earth, including the deep ocean (20), and is responsible for controlling a diverse range of cellular responses of different organisms (7). Until now, three families of photoreceptors (including cryptochromes, LOV, and BLUF) that use flavin chromophore as a cofactor have been identified (7, 21). Members of each family can respond to blue light at around 400 to 480 nm by using conserved photochemical changes (21). In particular, the BLUF domain acts as a blue light switch and responds to blue light by controlling specific enzyme activity or gene expression (22, 23). For example, a light-sensitive protein named AppA, binding the FAD chromophore in Rhodobacter sphaeroides, has been determined to regulate the expression of corresponding genes in response to light and dark stimuli; AppA correlates with the transcriptional suppressor PpsR in the dark, and this interaction is disrupted to activate downstream gene expression under blue light (23, 24).

Extensive studies have been performed to investigate light sensing or utilization of light by photosynthetic and nonphotosynthetic microorganisms existing in the photic zone. However, the existence and mechanisms of light sensing mediated by microbes living in the dark (e.g., the deep ocean) have been seldom discovered and reported. Surprisingly, recently evidence has started to accumulate that there are two forms of light distributed in the deep ocean: one is the downwelling light originating from the sun and stars, and it mainly exists in the mesopelagic region (150 to 1,000 m), dominated by blue-green light; the other is the bioluminescence generated by luminous organisms existing at all depths (20). As a result, the photosensing system of mesopelagic microorganisms has been pushed to its sensitivity limits to function in this extreme environment. For example, in the deep-sea hydrothermal vents, a nonphototrophic bacterium, Croceicoccus marinus OT19, that senses infrared light (wavelength, 940 nm) by the bacteriophytochrome has been reported (8). Compared to the hydrothermal vents, in the deep-sea cold seeps, short-wavelength and long-wavelength downwelling light are rapidly attenuated by seawater and gradually disappear along with the increasing depth, and the remaining light is mainly blue-green light (20). In addition, bioluminescent organisms such as lantern fish, mainly generating blue-green light, are also widely present in the deep-sea area (25). These kinds of faint blue-green light might provide a special light source to microorganisms living in the cold seeps for utilization through special photoreceptors. However, until now, microorganisms derived from the deep-sea cold seep that sense blue light have not been reported.

Here, a novel bacterial strain, *Spongiibacter nanhainus* CSC3.9, was enriched and purified from the deep-sea cold seep sample through a blue-light induction approach. In combination with genomic, proteomic, genetic, and biochemical methods, we detailed the mechanism of this novel strain that senses blue light by a BLUF-dependent pathway. Finally, the broad distribution of BLUF homologs in the genomes of marine microorganisms derived from diverse environments, including the cold seeps, was also analyzed and discussed.

## RESULTS

### Cultivation and characterization of a deeps-sea bacterium enriched by a blue light induction approach.

**FIG 1:**
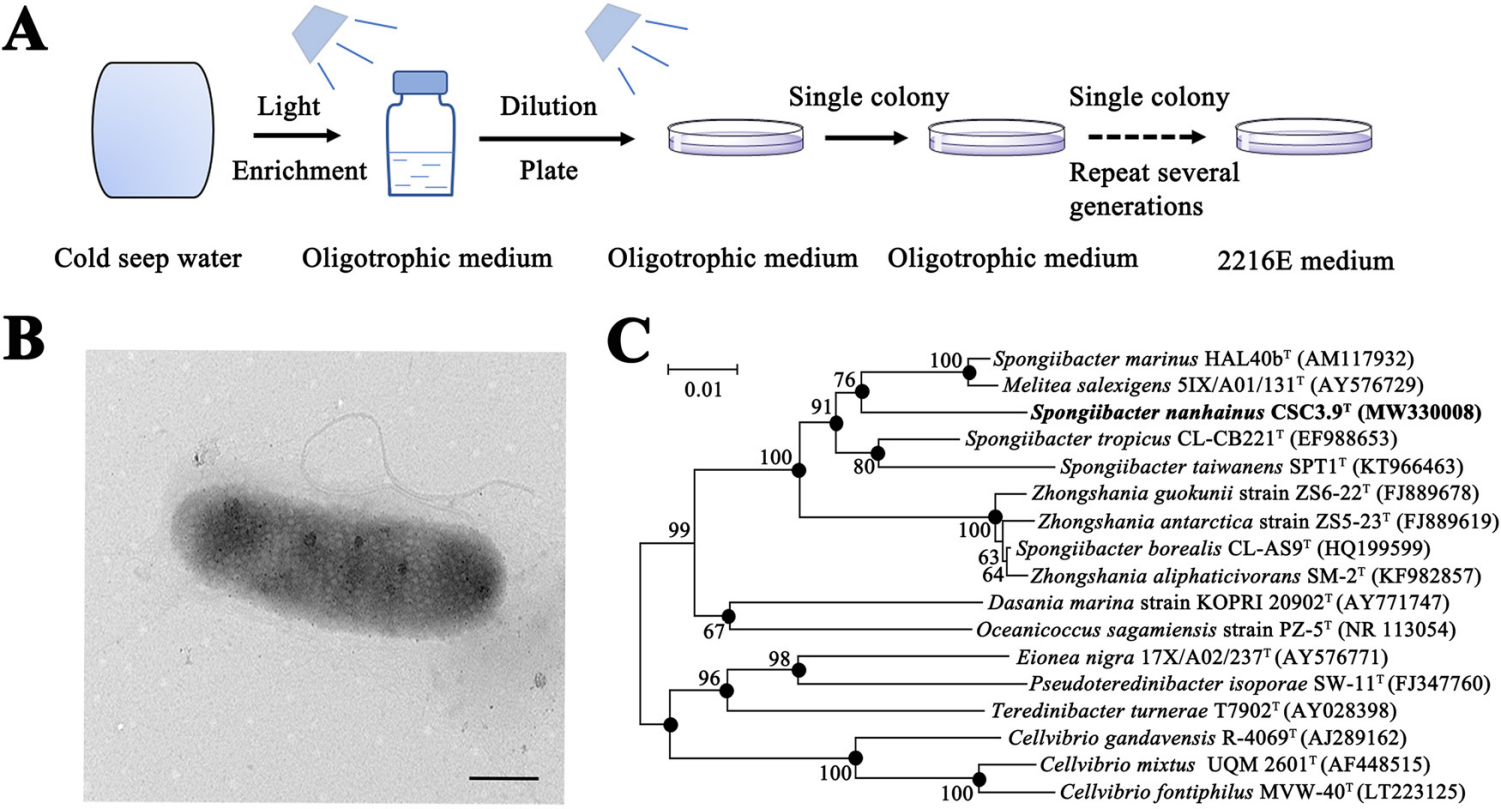
Cultivation, morphology, and phylogeny of the novel species *S. nanhainus* CSC3.9 isolated from a deep-sea cold seep. (A) Diagram of enrichment and isolation of strain CSC3.9 through a light induction approach. (B) Observation of the cell morphology of strain CSC3.9 through transmission electron microscopy. Bar, 500 nm. (C) Neighbor-joining phylogenetic tree based on 16S rRNA gene showing the positions of strain CSC3.9 and other closely related members. The phylogenetic tree was analyzed by three different algorithms (including neighbor-joining, maximum-likelihood, and maximum-parsimony methods). Bootstrap values (>60%) are shown by the side of the branch node circles for 1,000 replicates. The branch nodes with solid circles indicate they were recovered by three algorithms. Bar, 0.01 substitutions per nucleotide position.

To investigate the effects of light on the growth of deep-sea microorganisms, we enriched microorganisms with deep-sea cold seep samples in liquid oligotrophic medium (named SPG in this study) under different wavelengths of light and dark conditions, respectively. After 3 months of enrichment, each sample was diluted and plated on the solid SPG medium and incubated at 25°C for different periods. Surprisingly, only several white colonies were observed on the plate inoculated with the sample enriched under the blue light. After continuous purification and 16S rRNA sequencing confirmation, a pure bacterial strain named CSC3.9 was obtained (Fig. 1A). Cells of strain CSC3.9 were rods with single flagella (Fig. 1B). The phylogenetic analysis with 16S rRNA sequences of strain CSC3.9 together with other closely related strains was performed. The results revealed that strain CSC3.9 belonged to the class *Gammaproteobacteria* and was most closely related to Spongiibacter marinus strain HAL40b^T^ (95.75% similarity) (Fig. 1C), the type strain of genus *Spongiibacter* isolated from the marine sponge at the Sula Ridge (26). On the basis of taxonomic data collected in this study (shown in the supplemental material), we conclude that strain CSC3.9 is classified as the type strain of a novel species within the genus *Spongiibacter*, for which the name *Spongiibacter nanhainus* sp. nov. is proposed.

### Genetic and proteomic analyses of blue light sensing by *S. nanhainus* CSC3.9.

**FIG 2:**
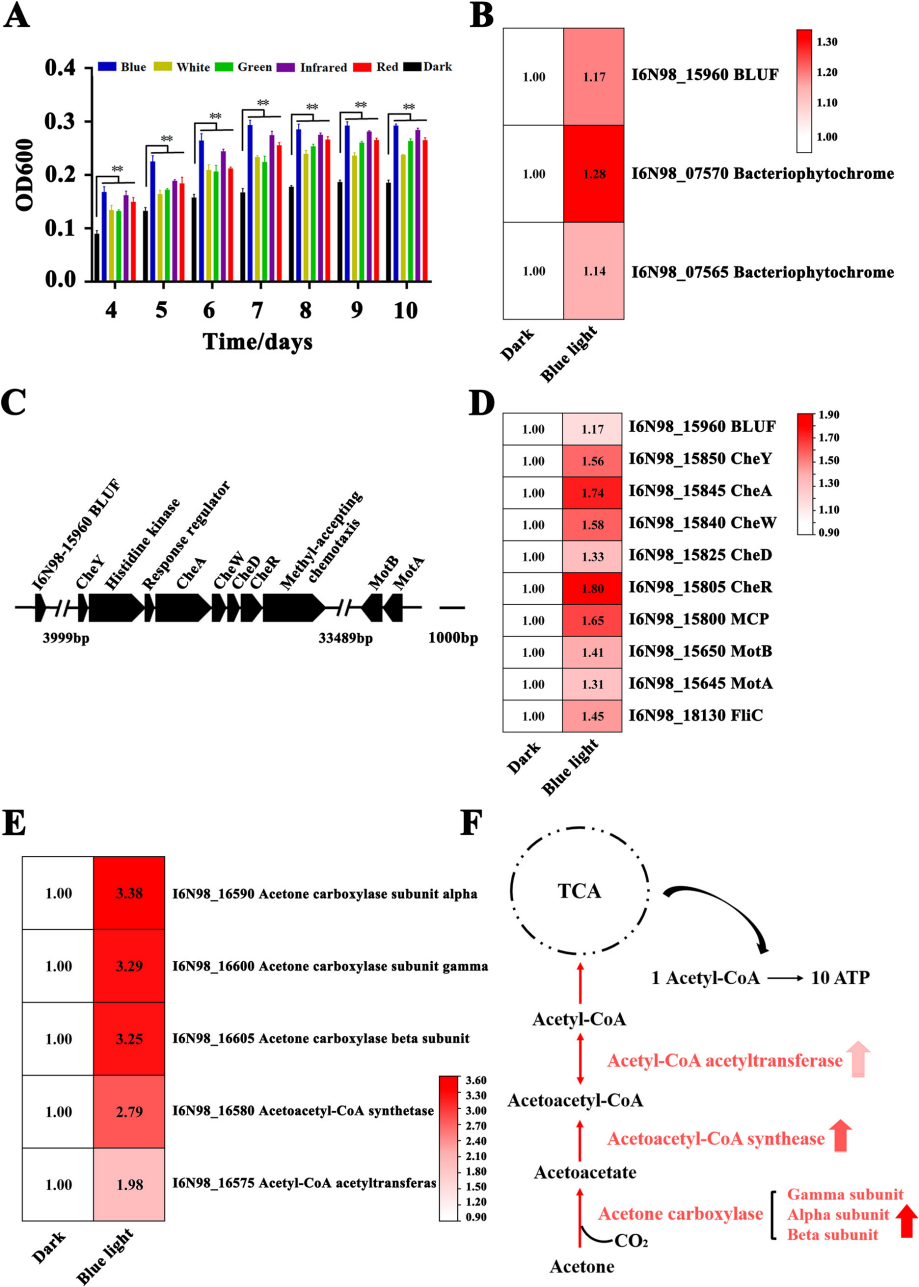
Proteomic determination of key factors responsible for blue light sensing and utilization in *S. nanhainus* CSC3.9. (A) Growth assays of strain CSC3.9 exposed to six different wavelength ranges of light. The means ± standard deviations (SD) from three experiments are shown. **, *P < *0.01 versus control. (B) Proteomics-based heat map showing all significantly upregulated proteins identified as blue light receptors (including two bacteriophytochrome-like proteins, I6N98_07570 and I6N98_07565, and one blue light sensor, BLUF, I6N98_15960) in strain CSC3.9 that were cultured under the blue light stimulation. (C) Arrangements of a putative gene cluster encoding proteins responsible for chemotaxis and flagellar stators in strain CSC3.9. The gene cluster was located immediately downstream of the gene encoding the blue light sensor, BLUF (I6N98_15960). The numbers 3,999 bp and 33,489 bp indicate the lengths of two omitted parts in this gene cluster. (D) Proteomics-based heat map showing all significantly upregulated proteins associated with chemotaxis in strain CSC3.9 cultured under blue light stimulation. (E) Proteomics based heat map showing all significantly upregulated proteins associated with intracellular acetone metabolism to produce acetyl-CoA in strain CSC3.9 cultured under blue light stimulation. (F) Scheme of acetone metabolism to produce acetyl-CoA for the TCA pathway. The protein names shown in this scheme are the same as those shown in panel E. The red up arrows indicate the expression of corresponding enzyme is significantly upregulated in strain CSC3.9 cultured under blue light stimulation.

Given that strain CSC3.9 was enriched and isolated from the sample exposed to the blue light, we further measured its growth status under luminous and dark conditions to confirm its blue light-sensing characteristic. Indeed, compared with other wavelengths of light, blue light stimulated better growth of strain CSC3.9 than dark and other luminous conditions (Fig. 2A). The annotation of the genome of strain CSC3.9 showed that it possessed genes encoding two bacteriophytochrome-like proteins (I6N98_07570 and I6N98_07565) and four putative blue light sensors (BLUF; I6N98_15960, I6N98_10885, I6N98_15525, and I6N98_16570). Altogether, these results strongly suggest that strain CSC3.9 senses and utilizes blue light.

To further explore the mechanism of light sensing by *S. nanhainus* CSC3.9, a proteomic assessment of strain CSC3.9 cultured under dark and blue light conditions was performed. The results of this assay revealed that the expression of 109 and 97 proteins (*P < *0.05) of strain CSC3.9 exposed to the blue light was significantly upregulated and downregulated compared to that under dark conditions. Of note, the expression of one blue light sensor (BLUF, I6N98_15960) and two bacteriophytochrome-like proteins (I6N98_07570 and I6N98_07565) all was evidently upregulated (Fig. 2B), indicating their roles in mediating blue light sensing in strain CSC3.9. On the other hand, the COG (Clusters of Orthologous Groups of proteins) functions of differentially expressed proteins identified with proteomic approach were classified and analyzed by NCBI database. The results showed that 18 COGs were related to cell motility and 29 COGs were related to signal transduction mechanisms (see Fig. S1 in the supplemental material), suggesting that the exposure of blue light is associated with the motility of strain CSC3.9, as shown in previous reports (15, 16). Coincidentally, there was a gene cluster encoding all essential components responsible for chemotaxis together with a gene pair encoding bacterial flagellar stator proteins (MotA and MotB) located immediately downstream of the BLUF (I6N98_15960)-encoding gene (Fig. 2C). Most importantly, the expression of all factors associated with chemotaxis and flagellar stator within the above-mentioned gene cluster and gene pair was significantly upregulated after strain CSC3.9 was exposed to blue light (Fig. 2D), further highlighting the key role of BLUF (I6N98_15960) in directing blue light sensing and motility in strain CSC3.9. Moreover, the expression of necessary components (including acetone carboxylase, acetyl-coenzyme A [CoA] acetyltransferase, and acetoacetyl-CoA synthetase) responsible for acetoacetyl-CoA production from acetone in strain CSC3.9 was all significantly upregulated after blue light treatment (Fig. 2E and F). It is known that the intracellular transformation from acetone to acetyl-CoA can effectively promote ATP production via the tricarboxylic acid (TCA) pathway (27, 28), which thereby facilitates the growth of strain CSC3.9, as shown in Fig. 2A.

10.1128/msystems.01279-21.2 FIG S1 COG analysis of proteomic differential proteins under the condition of blue light versus dark. Download FIG S1, DOCX file, 0.2 MB. Copyright © 2022 Shan et al. 2022 Shan et al. https://creativecommons.org/licenses/by/4.0/ This content is distributed under the terms of the Creative Commons Attribution 4.0 International license.

### Genetic determination of key factors responsible for blue light sensing in *S. nanhainus* CSC3.9.

**FIG 3:**
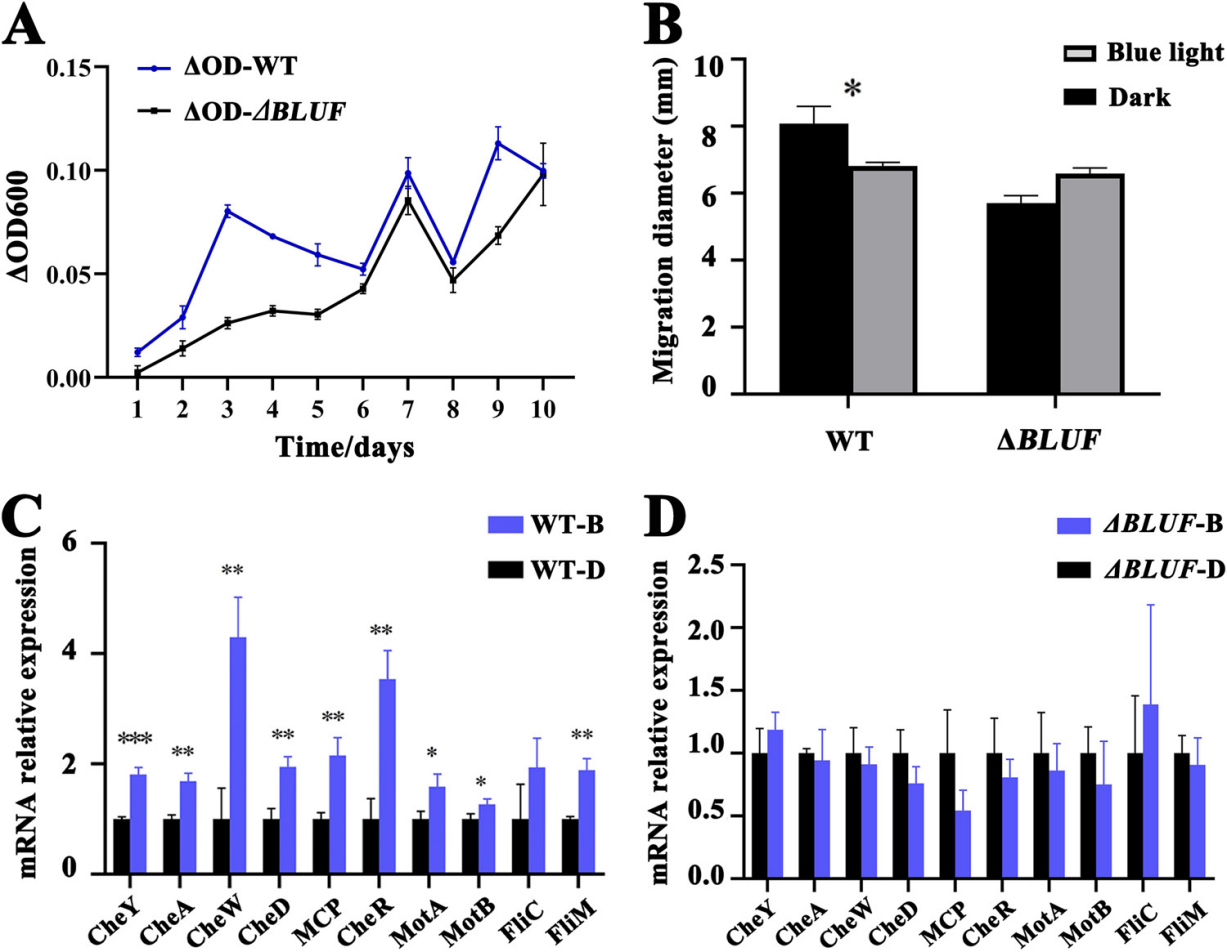
Genetic determination of BLUF as the essential factor directing blue light sensing in *S. nanhainus* CSC3.9. (A) Effects of blue light exposure on the growth of the wild type and *BLUF* deletion mutant of strain CSC3.9 when cultured under blue light and dark conditions. ΔOD indicates the differential OD_600_ values of cells (wild type or *BLUF* deletion mutant) grown under the blue light and dark conditions. (B) Measurement of migration zone diameters of the wild type and *BLUF* deletion mutant of strain CSC3.9 cultured under blue light and dark conditions. The assays were performed on a 0.3% semisolid agar plate containing 2216E medium. (C and D) qRT-PCR analysis of the expression of genes encoding chemotaxis-associated proteins in the wild type (C) and *BLUF* deletion mutant (D) of strain CSC3.9 cultured under blue light and dark conditions. In panel D, B indicates cells cultured under blue light condition and D indicates cells cultured under dark condition. The means ± SD from three experiments were shown. *, *P < *0.05; **, *P < *0.01; ***, *P < *0.001 versus the control.

Based on the proteomic result, we proposed that the blue light sensor (BLUF, I6N98_15960) and two bacteriophytochrome-like proteins (I6N98_07570 and I6N98_07565) determine light sensing in strain CSC3.9 (Fig. 2B). Next, we sought to thoroughly investigate the roles of these three key proteins identified in the proteomic analysis by creating gene knockouts *in vivo*. After much effort, a genetic operating system was successfully constructed for the first time in the genus *Spongiibacter*. Notably, the deletion of *BLUF* (*I6N98_15960*) resulted in slower growth of wild-type strain CSC3.9 than that of the *BLUF* deletion mutant (Δ*BLUF*) compared to the differential growth rates under dark and blue luminous conditions (Fig. 3A), confirming the key role of BLUF in mediating light sensing in strain CSC3.9, as shown in the proteomic analysis (Fig. 2B). Moreover, the motile capability of wild-type strain CSC3.9 was obviously weakened when comparing the migration distance in the soft agar plate under dark and blue lighting conditions (Fig. 3B). In contrast, the motile capability of the Δ*BLUF* strain was not affected when culturing the deletion mutant under dark and blue lighting conditions (Fig. 3B). We speculate that the ubiquitous distribution of light in the incubating equipment used in this study resulted in continuous tumble status for bacterial motility. As a result, bacterial cells lack the ability to migrate in a straight line, leading to the decline of the migration ability of wild-type strain CSC3.9. In contrast, the *BLUF* deletion mutant is unable to sense blue light, and the cells could keep their inherent motile capability on the soft agar plate, resulting in an obvious decrease of the motile distance of wild-type cells compared to that of the *BLUF* deletion mutant. The expression of genes within the gene pair and gene cluster encoding flagellar stators and proteins responsible for bacterial chemotaxis was simultaneously upregulated compared to the expression levels of the above-described genes of strain CSC3.9 cultured under blue light and dark conditions (Fig. 3C). On the contrary, the expression of these genes evidently did not change in the Δ*BLUF* mutant cultured under blue light and dark conditions (Fig. 3D). Taking these results together, we conclude that BLUF (I6N98_15960) is a key factor determining blue light sensing and motility in *S. nanhainus* CSC3.9.

On the other hand, the deletion of two genes encoding bacteriophytochrome-like proteins (I6N98_07570 and I6N98_07565) did not result in obvious effects on the growth of strain CSC3.9 cultured under blue light and dark conditions (Fig. S2). Moreover, the deletions of the two genes evidently did not change the expression of genes encoding flagellar stators and proteins responsible for chemotaxis of strain CSC3.9 cultured under blue light and dark conditions (Fig. S3A and B). Additionally, according to the main domains of these two bacteriophytochrome-like proteins predicted with reference to the SMART database (29) (Fig. S3C and 3D), neither of these two proteins contained a typical PAS-GAF-PHY photoreceptive region (30), indicating these two bacteriophytochrome-like proteins were not essential for strain CSC3.9 to sense blue light.

10.1128/msystems.01279-21.3 FIG S2 Effects of blue light exposure on the growth of the wild-type (WT) and two bacteriophytochromes-like genes (I6N98_07570 and I6N98_07565) of a deletion mutant of strain CSC3.9 when cultured under blue light and dark conditions. Download FIG S2, DOCX file, 0.1 MB. Copyright © 2022 Shan et al. 2022 Shan et al. https://creativecommons.org/licenses/by/4.0/ This content is distributed under the terms of the Creative Commons Attribution 4.0 International license.

10.1128/msystems.01279-21.4 FIG S3 Genetic analysis and structural prediction of two bacteriophytochrome-like proteins identified in strain CSC3.9 via proteomic assays. Download FIG S3, DOCX file, 0.1 MB. Copyright © 2022 Shan et al. 2022 Shan et al. https://creativecommons.org/licenses/by/4.0/ This content is distributed under the terms of the Creative Commons Attribution 4.0 International license.

### *In vitro* functional assays of BLUF of *S. nanhainus* CSC3.9.

**FIG 4:**
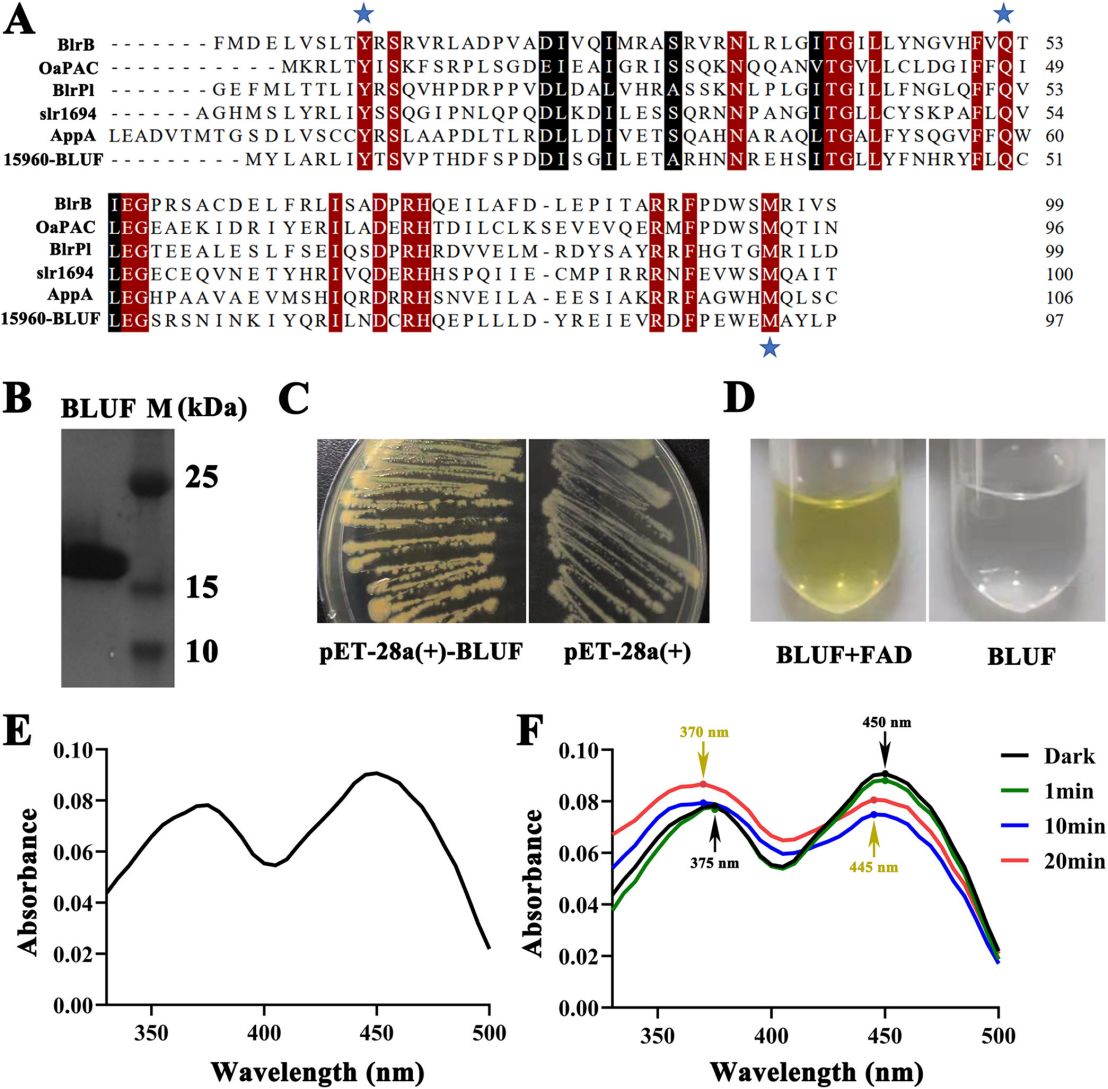
*In vitro* functional assay of BLUF mediating blue light sensing. (A) Sequence alignment of BLUF of strain CSC3.9 with other closely related homologs deposited in the Protein Data Bank. (B) SDS-PAGE analysis of BLUF purified from E. coli BL21(DE3) cells. M, protein marker. (C) Observation of colony color of E. coli BL21(DE3) cells with [pET-28a(+)-BLUF] or without [pET-28a(+)] overexpressed BLUF of strain CSC3.9. (D) Observation of the color change of purified BLUF supplemented with FAD left unsupplemented. (E) Absorption spectrum of purified BLUF bound with FAD. (F) Absorption spectra of BLUF (0.5 mg/ml) after being exposed to dark and white light (450 μmol m^−2^ s^−1^) for 1 min, 10 min, and 20 min.

Given that BLUF is the most essential factor controlling blue light sensing for strain CSC3.9, we next sought to verify its function in blue light sensing *in vitro*. First, a sequence alignment of BLUF (I6N98_15960) of strain CSC3.9 and six BLUFs having independent experimental models in the Protein Data Bank was performed (Fig. 4A). The alignment results showed that residues clustered around the flavin chromophore, and residues (including tyrosine, glutamine, and methionine) closely related to the function of the photocycle (7, 31, 32) were highly conserved, suggesting BLUF of strain CSC3.9 is indeed a member of the BLUF family proteins. Next, to determine the blue light-sensing function of BLUF (I6N98_15960) *in vitro*, BLUF of strain CSC3.9 was overexpressed and purified in E. coli BL21 cells (Fig. 4B). Interestingly, E. coli BL21 cells expressing BLUF showed a distinct yellow color compared to the original gray color of E. coli BL21 cells (Fig. 4C), indicating BLUF could specifically bind to its cofactor, FAD, produced by E. coli. BLUF changed to colorless after removing the bound FAD (Fig. 4D). BLUF showed two maximum absorption spectrum peaks at 375 nm and 450 nm (Fig. 4E). Of note, after a short-term (10 min or 20 min) exposure to white light (450 μmol m^−2^ s^−1^) excitation, there was a spectrum shift, showing a new absorption peak at 370 nm and 445 nm (Fig. 4F, Fig. S4).

10.1128/msystems.01279-21.5 FIG S4 Differential spectrum between treatments of BLUF with dark and irradiation for 1 min, 10 min, or 20 min by white light at an intensity of 450 μ mol m^−2^ s^−1^. Download FIG S4, DOCX file, 0.1 MB. Copyright © 2022 Shan et al. 2022 Shan et al. https://creativecommons.org/licenses/by/4.0/ This content is distributed under the terms of the Creative Commons Attribution 4.0 International license.

**FIG 5:**
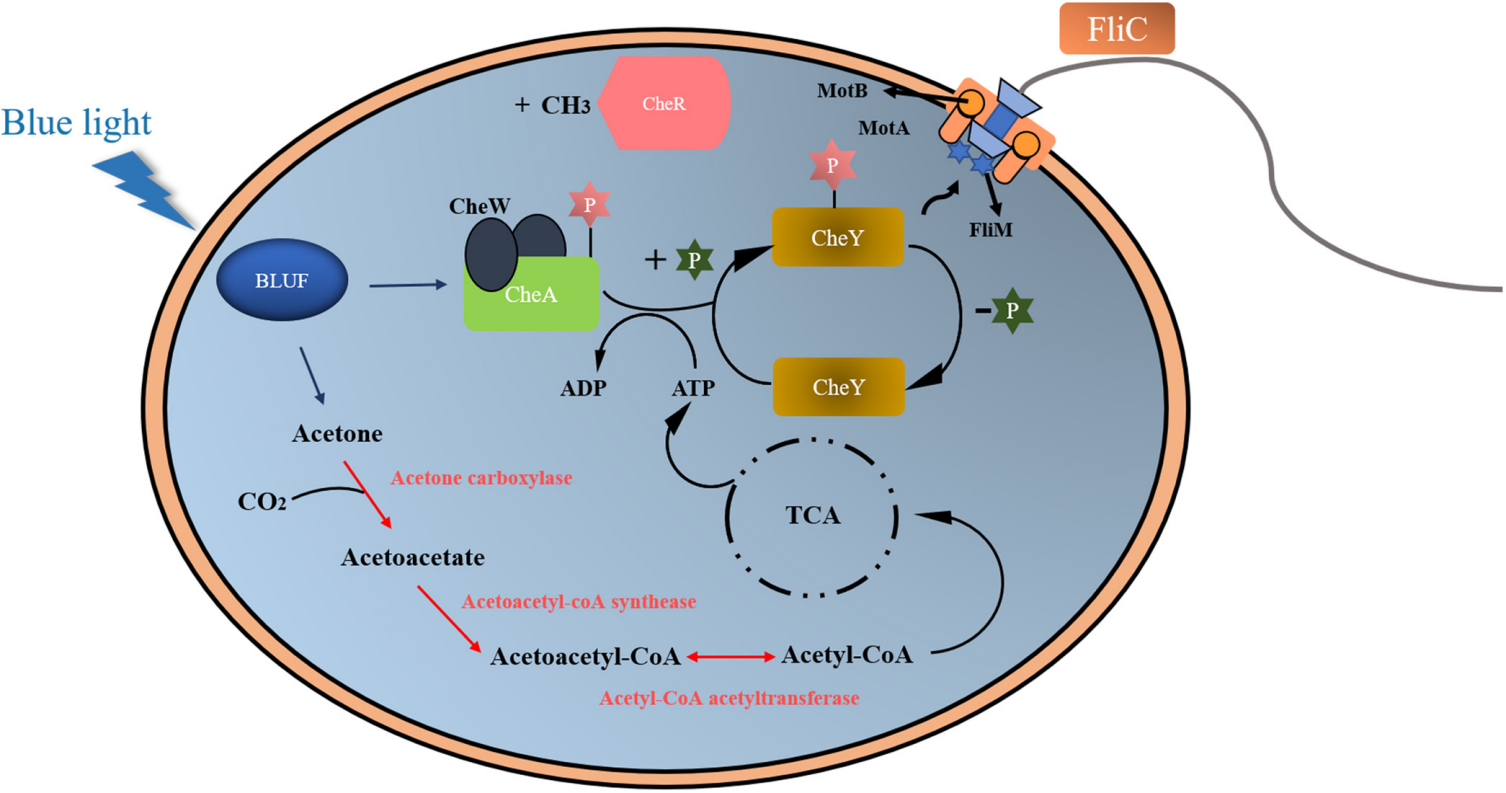
Proposed model of blue light sensing and utilization directly mediated by BLUF of *S. nanhainus* CSC3.9. Briefly, as a light receptor, BLUF senses the blue light and activates the acetone metabolism to produce acetyl-CoA for the TCA pathway, which generates enough energy for motility regulation and growth promotion. CheA, histidine protein kinase; CheY, response regulatory protein; CheR, methyltransferase; P, phosphorylation; CH3, methyl.

Based on the combination of proteomic, genetic, and *in vitro* data on blue light sensing and utilization conducted by *S. nanhainus* CSC3.9, we proposed a novel BLUF-dependent downstream response pathway and growth promotion system (Fig. 5). Briefly, blue light, as an adaptive signal of strain CSC3.9, was sensed and transmitted by BLUF to the components (including CheA, CheW, CheY, and CheR) responsible for bacterial phototaxis-related motility (33, 34). On the other hand, blue light stimulates the pathway responsible for the production of acetyl-CoA from acetone metabolism, thereby generating more energy through the TCA pathway (35) to support bacterial motility and growth.

### Wide distribution of BLUF homologs in marine microorganisms.

**FIG 6:**
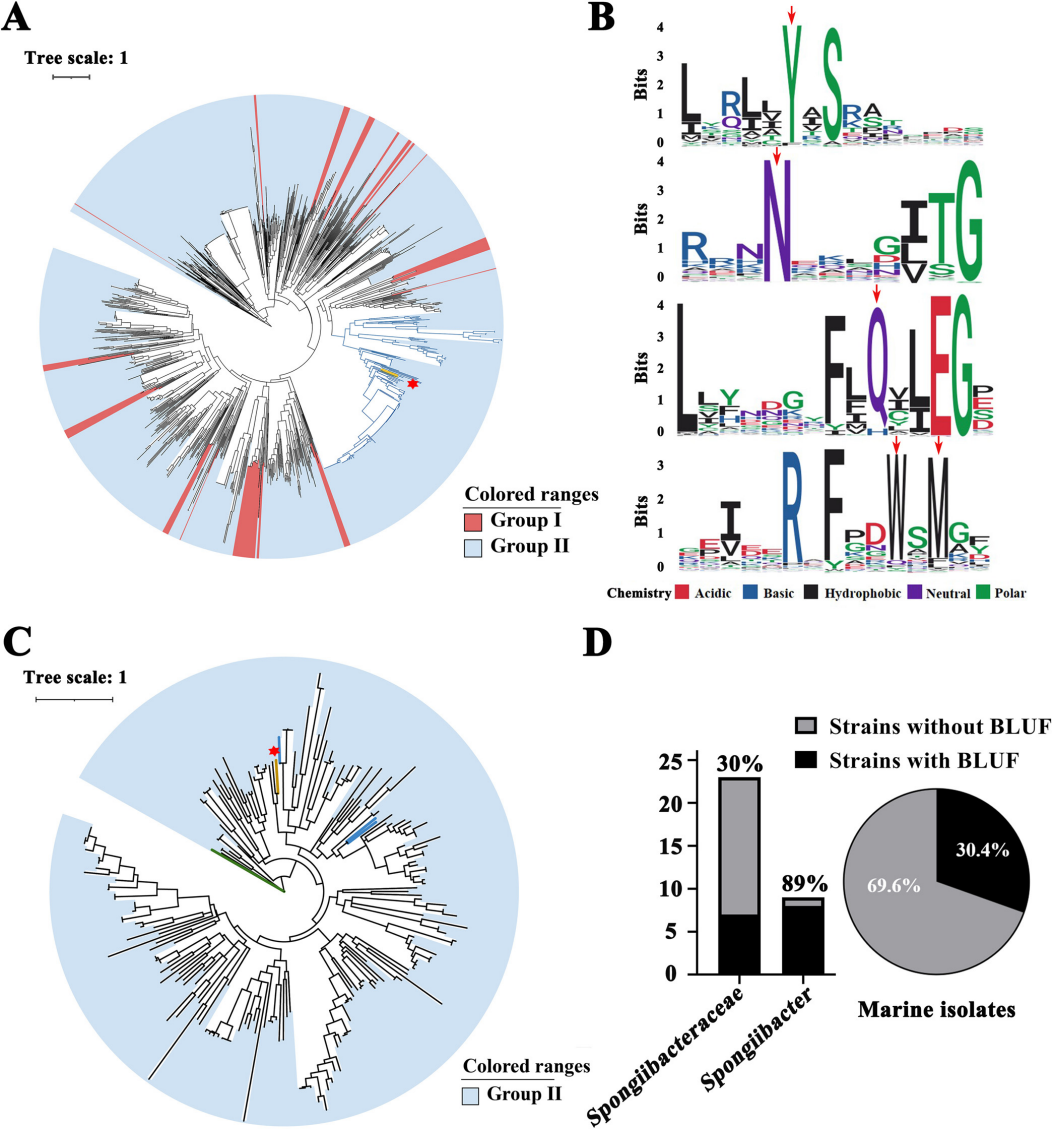
Alignment and phylogenetic analyses of BLUF homologs distributed in marine microorganisms. (A) The consensus phylogenetic tree of 1,049 BLUF homologs (including BLUF of strain CSC3.9) obtained from the cultured marine bacterial strains. The clade marked with red and blue backgrounds indicate that BLUF proteins belonged to group I and group II. BLUF (I6N98_15960) of the strain CSC3.9 branch was labeled yellow with a red asterisk. (B) Logo diagram of conserved motifs and amino acid residues (marked with a red down arrow) based on 1,049 BLUF homologs shown in panel A. The level of preference/depletion is shown height proportional to the scale. (C) The consensus phylogenetic tree of BLUF homologs obtained from the metagenomic data of microbes derived from two marine cold seeps. Clades marked with yellow (BLUF I6N98_15960 of strain CSC3.9), blue, and green represent three types of BLUF homologs possessing verified blue light-sensing functions. (D) The proportion of BLUF homologs identified in the genomes of cultured strains belonging to the family *Spongiibacteraceae* and the genus *Spongiibacter*.

To explore homologs of BLUF, we searched for all assembled genomes of cultured marine microorganisms deposited in the IMG database. The results revealed that among the 2,322 genomes of marine isolates deposited in the database there were 1,048 BLUF homologs, indicating BLUF was present in most phyla of marine bacteria (Table S5). Based on the reported classification criterion of BLUF homologs, the ones having more than 200 amino acid residues were defined as group I, and those possessing amino acid residues less than 200 were defined as group II (20). The SMART database modular architecture prediction showed that 92.7% of these 1,048 BLUF homologs (including the BLUF of CSC3.9) identified in marine microorganisms belonged to group II (Fig. 6A). Moreover, amino acid residues (including tyrosine, asparagine, glutamine, tryptophan, and methionine) critical to the photocycling and photodynamics in these 1,048 BLUF homologs (7, 36, 37) were highly conserved (Fig. 6B), which might ensure the reliable blue light-sensing functions of BLUF homologs of bacteria existing in various habitats.

10.1128/msystems.01279-21.10 TABLE S5 Distribution of BLUF gene identified in different phyla of isolated marine microorganisms deposited in the IMG database. Download Table S5, DOCX file, 0.02 MB. Copyright © 2022 Shan et al. 2022 Shan et al. https://creativecommons.org/licenses/by/4.0/ This content is distributed under the terms of the Creative Commons Attribution 4.0 International license.

It is notable that there were 212 *BLUF* genes (which were all classified as group II) identified in the metagenomes of microorganisms living in the cold seeps located at Baltimore Canyon and Hudson Canyon (depth of 540 m, U.S. Atlantic Margin), indicating that marine bacteria existing in the marine cold seeps also had potential to sense blue light (Fig. 6C).

## DISCUSSION

It is notable that microbes in the deep ocean represent a large portion of the biosphere, and deep-sea environments have been a prominent source of new lineages in the tree of life (38, 39). Even though microbes in the deep ocean have extensively expanded the tree of life, there are still many additional uncultured lineages left to be explored. Therefore, it is urgent that we develop more efficient cultivation strategies to obtain the uncultured microbial majority. In the present study, a deep-sea cold seep bacterium, *S. nanhainus* CSC3.9, was isolated through an innovative approach: enrichment under blue light stimulus (Fig. 1A). Based on the physiological and phylogenetic analyses, strain CSC3.9 was proposed to be a novel species within the genus *Spongiibacter* (Fig. 1C). Until now, most *Spongiibacter* members were isolated from the ocean surface (40–42); in contrast, *S. nanhainus* CSC3.9 was the first *Spongiibacter* strain isolated from deep-sea cold seep water. Therefore, light induction should be considered a novel approach to enrich and isolate more uncultured microorganisms from the deep sea. Recently, more and more innovative methods have been developed to enrich and isolate difficult-to-cultivate microbes from the deep ocean, such as the cultivation of the first free-living representative of *Candidatus* Izemoplasma through a DNA degradation-driven strategy (38), the isolation of a novel species of deep-sea *Bacteroidetes* by a polysaccharide degradation method (43), and the decade-long isolation of an Asgard archaeon related to Lokiarchaeota via a cocultivation approach (44). It is reasonable to propose that the growing interest in and need for efficient cultivation strategies will lead to many rapid methodological and technological advances in the near future.

The most impressive trait of *S. nanhainus* CSC3.9 is its ability to sense blue light (Fig. 2 and 3). As a deep-sea bacterium, it evolves a property that senses light signals. Actually, more and more evidence shows that the deep ocean possesses some special light sources (20, 25). Among these lights, bioluminescent emissions (blue-green range of the spectrum) from animals are the predominant source of light (20, 25). A record containing 17 years of video observations by remotely operated vehicles during surveys off the California coast from the surface down to 3,900 m depth shows that 76% of oceanic marine organisms observed in deep waters offshore of California have the capability of bioluminescence (25). Given that the deep ocean is the largest habitat on earth by volume, bioluminescence can certainly be said to be a major ecological trait on earth (25). Therefore, it is not surprise that a lot of microorganisms living in the deep sea have evolved different strategies to utilize light as a beneficial complement for growth support and adaptation to the harsh condition. Notably, different wavelengths of light could stimulate the growth of *S. nanhainus* CSC3.9 (Fig. 2A), suggesting other types of light (including green, red, and infrared) exist in the deep sea, and microorganisms (like strain CSC3.9) might evolve a corresponding apparatus for light sensing. In the deep-sea cold seep where strain CSC3.9 was isolated, a lot of mussels and shrimps were found (38); however, it is not known whether they have bioluminescence capabilities. Corresponding apparatus and techniques should be developed to detect and record the bioluminescence level of cold seeps in the future, which will be helpful for researchers to understand the light-sensing mechanisms of microbes (e.g., *S. nanhainus* CSC3.9) in the deep biosphere.

In combining genomic, proteomic, genetic, and biochemical methods, we convincingly determine that the photoreceptor BLUF directs blue light sensing in *S. nanhainus* CSC3.9 (Fig. 2 and 4). Moreover, BLUF also changed the motile capability of strain CSC3.9 under the exposure of blue light (Fig. 3 and 4). Actually, the downwelling light and bioluminescence existing in the deep sea are mainly blue-green light (20, 25). Therefore, the blue light-sensing capability of *S. nanhainus* CSC3.9 endows this bacterium with an advantage to promptly seek the light source for its better growth. Indeed, blue light is a kind of efficient spectrum that is broadly utilized by the majority of photosynthetic and nonphotosynthetic organisms. Four blue light photoreceptors, including cryptochromes, LOV, PYP, and BLUF, have been discovered in nonphotosynthetic bacteria (7, 21). Among these photoreceptors, BLUF is broadly adopted by bacteria to sense blue light in nature (7). Our investigation based on the public database shows that BLUF homologs broadly distribute in the marine environments, including cold seep microorganisms (Fig. 6A to C). Thirty percent of genomes belonging to the *Spongiibacteraceae* deposited in the public database possess genes encoding BLUF homologs, and 8 of the 9 strains of genus *Spongiibacter* contained BLUF protein in their genomes (Fig. 6D). The ubiquitous distribution of BLUF homologs in marine microorganisms suggests the existence of light sensing in other nonphototrophic bacteria in different ocean environments.

In summary, our successful cultivation of a novel *Spongiibacteraceae* bacterium from the deep-sea cold seep via a light induction approach has demonstrated that enrichment and isolation of microbes under luminous conditions from the deep ocean should be an innovative method to obtain more uncultured microorganisms. This study extends our understanding of the unique biology of a novel bacterial strain toward light sensing and highlights that deep-sea bacteria may adopt many unknown pathways to adapt to the deep-sea harsh conditions.

## MATERIALS AND METHODS

### Sampling and culture conditions.

The water samples were collected by RV *KEXUE* from a cold seep in the South China Sea (119°17′04.956″E, 22°06′58.384″N) at a depth of approximately 1,121 m in September 2017. The samples, diluted by sterile seawater, were inoculated at 28°C and exposed to different wavelengths of 6-W fluorescence light (including white, wavelength at 400 to 760 nm [450 μmol m^−2^ s^−1^]; blue, wavelength at 465 to 470 nm [20 μmol m^−2^ s^−1^]; green, wavelength at 520 to 525 nm [90 μmol m^−2^ s^−1^]; red, wavelength at 620 to 625 nm [80 μmol m^−2^ s^−1^]; infrared, wavelength at 940 nm [5 μmol m^−2^ s^−1^]) or under a dark condition for 3 months in oligotrophic (SPG) medium containing (per liter seawater) 6.5 g PIPES (C_8_H_18_N_2_O_6_S_2_), 25 g NaCl, 2.7 g MgSO_4_·7H_2_O, 4.3 g MgCl_2_·6H_2_O, 0.25 g NH_4_Cl, 0.5 g KCl, 0.14 g CaCl_2_, 0.14 g K_2_HPO_4_·3H_2_O, 0.002 g Fe(NH_4_)_2_(SO_4_)_2_·6H_2_O, pH 6.8. After the enrichment, the samples were plated on the agar plate containing SPG medium. The visible colonies on the plate were further purified by repeatedly restreaking on the agar plate until the pure culture was obtained. The rejuvenated strains were reinoculated in 2216E medium (5 g tryptone and 1 g yeast extract in 1 liter seawater) to promote the growth of strain CSC3.9 and stored in the medium containing 50% (vol/vol) glycerol at −80°C.

### TEM observation.

Cell morphology of strain CSC3.9 was examined by transmission electron microscopy (TEM) with a JEOL JEM 12000 EX (equipped with a field emission gun) at 100 kV. Briefly, the liquid culture of strain CSC3.9 was washed with 10 mM phosphate-buffered saline (PBS, pH 7.4) and centrifuged at 5,000 × *g* for 5 min. The copper grid coated with carbon film then was immersed in bacterial suspension for 20 min, washed with distilled water for 5 min, dried at room temperature for 3 h, and then observed by TEM.

### Physiological and chemotaxonomic assays of strain CSC3.9.

The pH, temperature, and NaCl ranges and other optimum conditions for the growth of strain CSC3.9 were tested and indicated by the OD_600_ value in 2216E liquid culture. For both growth and characteristic detection, one-tenth 2216E liquid medium containing different electron donors and carbon sources (including glucose, sucrose, fructose, galactose, maltose, xylose, inositol, citrate, salicylic acid, succinate, pyruvate, arabinose, rhamnose, xylan, mannose, glycerol, and acetate) at 10 mM was used to detect the utilization by strain CSC3.9. The growth under different conditions was checked by OD_600_ value and used to determine the proper electron donor and carbon source of strain CSC3.9. Similarly, the OD_600_ value of strain CSC3.9 was also used to determine the growth status of this bacterium cultured under different wavelengths of light and dark conditions.

### 16S rRNA phylogenetic analysis of strain CSC3.9.

The 16S rRNA sequences of strain CSC3.9 and other related taxa were used for phylogenetic analysis derived from NCBI GenBank. MEGA version 6.0 software was used for phylogenetic analysis. The phylogenetic tree was completed by the minimum-evolution, maximum-likelihood, and neighbor-joining methods. The bootstrap values after 1,000 repetitions were marked above or below the branch.

### Genomic sequencing and analysis.

To sequence the whole genome, we extracted the total chromosomal DNA of strain CSC3.9. The DNA library was prepared and sequenced using the link-sequencing kit (SQK-LSK109), and the sequencing was performed with Minknown software v1.4.2 (Oxford Nanopore Technologies) using a Flol-Min106 VR9.4 flow cell. The whole genome was sequenced using Illumina MiSeq sequencing platforms (San Diego, CA) and Oxford Nanopore Minion (Oxford, United Kingdom) and further genome assembly using reads from both platforms. Base-calling was carried out by Albacore software v2.1.10. For quality control and downstream analysis purposes, Nanopore reads were processed using the PorreTools toolkit, and Canu version 1.8 (45) was used for assembling filtered reads. The whole genome was eventually assembled into a single contig and manually cycled by removing overlapping ends. For detection of contamination, 16S rRNA Sanger sequencing was performed prior to genome sequencing. The BUSCO assessment was used to test the completeness of the genome. Seven databases were used to predict gene functions. They were COG (Clusters of Orthologous Groups), NR (Non-Redundant Protein Database databases), GO (Gene Ontology), KEGG (Kyoto Encyclopedia of Genes and Genomes), TCDB (Transporter Classification Database), and Swiss-Prot. A whole-genome BLAST search (E value less than 1e^−5^, minimal alignment length percentage larger than 40%) and annotation were performed against these seven databases.

### Proteomic analysis.

Proteomic analysis was carried out by PTM Biolab, Inc. (Hangzhou, China), and followed procedures described previously (46). Briefly, strain CSC3.9 was incubated under dark and blue light conditions for 7 days, and cellular proteins were extracted and quantified after lysis in the buffer (containing 8 M urea, 1% protease inhibitor cocktail). After trypsin digestion, peptide was desalted by a Strata X C_18_ SPE column (Phenomenex) and vacuum dried. Peptide was reconstituted in 0.5 M triethylammonium bicarbonate and processed according to the manufacturer’s protocol for the TMT kit/iTRAQ kit. The tryptic peptides were fractionated into fractions by high-pH reverse-phase high-performance liquid chromatography using a Thermo Betasil C_18_ column (5-μm particles, 10-mm inner diameter, 250-mm length). The peptides were subjected to nanospray ionization source followed by tandem mass spectrometry (MS/MS) in Q Exactive Plus (Thermo, USA) coupled online to ultrahigh-performance liquid chromatography. The resulting MS/MS data were processed using Maxquant search engine (v.1.5.2.8). For protein quantification, a protein should contain at least two unique peptides. The ratio of interesting proteins in the blue light group/dark group was weighted and normalized to the median ratio in Mascot. Analysis of the differentially expressed proteins was performed using HemI software (47).

### Construction of deletion mutants of strain CSC3.9.

Homologous recombination techniques were used to generate knockout mutations in strain CSC3.9 according to the method described previously (46, 48, 49), with some modifications. To delete the *BLUF* gene (locus tag I6N98_15960), the upstream and downstream flanking regions of *BLUF* were amplified from the wild-type strain CSC3.9 genome using primer pairs BLUFup-F/BLUFup-R and BLUFdown-F/BLUFdown-R (see Table S3 in the supplemental material), respectively. The upstream and downstream PCR products were purified, combined, and used as the templates for an overlap extension PCR of KO-BLUF using primers BLUFup-F/BLUFdown-R. The KO-BLUF fragment was purified and inserted into the suicide vector pEX-18Gm via a ClonExpress ultra one-step cloning kit (Vazyme, China). The resulting plasmid (pEX-18Gm-KO-*BLUF*) was transformed sequentially into E. coli DH5α, E. coli SY327, and E. coli S17-1 using the CaCl_2_ method. Mating between strain CSC3.9 and E. coli S17-1 containing pEX-18Gm-KO-*BLUF* was cultured at 28°C for at least 72 h. Single-event recombinant strains were selected on 2216E agar plates supplemented with kanamycin (100 μg/ml) and gentamicin (25 μg/ml). Colonies containing pEX-18Gm-KO-*BLUF* were isolated on 2216E agar plates supplemented with 15% sucrose and kanamycin (100 μg/ml). Putative mutants were checked by PCR with primers BLUFup-F/BLUFdown-R. The same methods were used to construct other mutant strains using the corresponding primers listed in Table S3.

10.1128/msystems.01279-21.6 TABLE S1 Differential physiological and phenotypic characteristics of the strain CSC3.9 and the type strain HAL40b^T^. Download Table S1, DOCX file, 0.03 MB. Copyright © 2022 Shan et al. 2022 Shan et al. https://creativecommons.org/licenses/by/4.0/ This content is distributed under the terms of the Creative Commons Attribution 4.0 International license.

10.1128/msystems.01279-21.7 TABLE S2 Main fatty acids (%) of the strain CSC3.9^T^ compared with its closest type strain, HAL40b^T^. Download Table S2, DOCX file, 0.02 MB. Copyright © 2022 Shan et al. 2022 Shan et al. https://creativecommons.org/licenses/by/4.0/ This content is distributed under the terms of the Creative Commons Attribution 4.0 International license.

10.1128/msystems.01279-21.8 TABLE S3 Strains, plasmids, and primers used for vector construction. Download Table S3, DOCX file, 0.03 MB. Copyright © 2022 Shan et al. 2022 Shan et al. https://creativecommons.org/licenses/by/4.0/ This content is distributed under the terms of the Creative Commons Attribution 4.0 International license.

### Motile capability assays of strain CSC3.9 under blue light exposure.

To analyze the motile behavior of strain CSC3.9 and the *BLUF* deletion mutant strain under the blue light and dark conditions, 5 μl of bacterial cells cultured in the dark to logarithmic phase was dropped in the center of a 2216E semisolid plate containing 0.3% agar. After drying, the plates were cultured under blue light or dark conditions. After culturing for 5 days, the colony diameter was measured to indicate the motile capability of bacterial cells, and the final motile distance was based on five biological replicates.

### qRT-PCR.

To perform the quantitative real-time PCR assay (qRT-PCR) assays, the wild-type strain CSC3.9 and corresponding gene deletion mutants were inoculated in the 2216E medium under the blue light and dark conditions until the OD_600_ value was about 0.6. Thereafter, bacterial cells were collected by centrifugation at 6,000 × *g* for 15 min. Total RNAs were then extracted by TRIzol reagent (Solarbio, China) and reverse transcribed into cDNA. The completion of qRT-PCR was checked by using the SYBR green real-time PCR master mix (Toyobo Co., Ltd., Osaka, Japan), and the transcription levels of different genes were checked by using the ABI 7900 real-time PCR system (Applied Biosystems, Foster City, CA, USA). 16S rRNA was used as an internal reference gene. The relative gene expression was calculated using the 2^−ΔΔ^*^CT^* method. Specific primers for the 16S rRNA and the genes within chemotaxis gene clusters were designed by Primer 6.0, as shown in Table S4. Three biological replicates and three technical replicates were performed in all the qRT-PCR assays.

10.1128/msystems.01279-21.9 TABLE S4 Primers used for qRT-PCR. Download Table S4, DOCX file, 0.02 MB. Copyright © 2022 Shan et al. 2022 Shan et al. https://creativecommons.org/licenses/by/4.0/ This content is distributed under the terms of the Creative Commons Attribution 4.0 International license.

### Protein expression, purification, and activity assay.

The *BLUF* gene was amplified from the genome of strain CSC3.9 using the primers 28aBLUF-F/28aBLUF-R, shown in Table S3. The PCR product was purified, digested, and ligated into the expression vector pET-28a(+) (Solarbio, China). Recombinant plasmid was transformed into BL21(DE3) E. coli cells. Cells were grown aerobically at 37°C in LB medium (10 g tryptone, 5 g yeast extract, and 10 g NaCl in 1 liter distilled water) supplemented with kanamycin at a final concentration of 100 μg/ml. Protein expression was induced at an OD_600_ around 0.6 with 0.1 mM isopropyl-1-thio-β-d-galactopyranoside, and the cells were cultured for a further 20 h at 16°C. Cells were harvested by centrifugation (8,000 × *g*, 20 min, 4°C), resuspended in the buffer A (containing 50 mM Tris-HCl, 150 mM NaCl, pH 8.0), and subjected to sonication. The lysate was centrifuged and filtered, and FAD was added to the supernatant at a final concentration of 30 μM and incubated for another 1 h on ice. The treated supernatant was loaded onto a 5-ml HisTrapTM HP column (GE Healthcare, USA), and the proteins were eluted with buffer B (containing 500 mM imidazole, 150 mM NaCl, 50 mM Tris-HCl, pH 8.0). The fractions were then collected and dialyzed at 4°C overnight against 5 liters of buffer A. The purified BLUF (I6N98_15960) was dialyzed in the PBS buffer (10.1 mM Na_2_HPO_4_, 1.8 mM KH_2_PO_4_, 1.8 mM KCl, and 140 mM NaCl at pH 7.3) supplemented with 6 M guanidine hydrochloride overnight at 4°C. Denatured proteins resulted in a complete release of FAD. The denatured proteins were then dialyzed against the PBS buffer supplemented with 3 M guanidine hydrochloride, 30 μM FAD, and 0.1 M arginine hydrochloride for 8 h at 4°C. The sample was further dialyzed against the PBS buffer supplemented with 1.5 M guanidine hydrochloride, 30 μM FAD, and 50 mM arginine hydrochloride for 8 h at 4°C and finally dialyzed against the PBS buffer overnight at 4°C. The dialyzed samples were centrifuged at 5,000 × *g* for 20 min to remove denatured proteins. The protein was concentrated with a 3-kDa ultrafiltration centrifuge tube to a final concentration of 0.5 mg/ml. Protein purity and yield were determined using SDS-PAGE and a NanoPhotometer spectrophotometer. Holo-protein solutions containing 0.5 mg/ml BLUF in the PBS buffer were taken into the 1.5 ml-Eppendorf tubes and exposed to white light (450 μmol m^−2^ s^−1^) for 1 min, 10 min, and 20 min. Finally, the absorbance spectra of BLUF solution were measured at 330 to 500 nm with an interval of 5 nm using a multidetection microplate reader (Infinite M1000 Pro; Tecan, Switzerland).

### Identification of BLUF homologs in marine bacterial genomes.

To perform the alignment analysis of BLUF homologs, 2,322 genomes of cultured bacterial strains from different marine environments were obtained from the Integrated Microbial Genomes (IMG) database by genome searching (50). Using the advanced search function in the database, 1,048 BLUF homologs were selected from 2,322 genomes. Multiple EM for Motif Elicitation (MEME) suite (http://meme-suite.org) was used to scan the 1,048 BLUF amino acid sequences for conserved motif and amino acid sites (51). By searching for the cold seep metagenome data uploaded to the IMG database, we acquired 212 BLUF homologs from bacterial metagenomes of methane seeps at Baltimore Canyon and Hudson Canyon (depth of 540 m, U.S. Atlantic Margin). For phylogenetic analysis, amino acid sequences of BLUF from marine and cold seep environments were extracted from IMG databases. In addition, we obtained 4 BLUF amino acid sequences with clear structure in the Pfam database (V5VB82, Q8DMN3, P74295, and A6T8V8), which were used to construct the phylogenetic tree of BLUF homologs derived from cold seep metagenomes. After sequence alignment, IQ-TREE online software was used to construct the maximum likelihood phylogenetic tree using LG+G4+F (-BB 1000) as a model (52). The sequence alignments for all trees were calculated using the MEGA X software with Clustal W/MUSCLE program (53). All trees were visualized decorated using iTOL (v5) (54).

### Data availability.

The complete genome sequence of *Spongiibacter nanhainus* CSC3.9 has been deposited in GenBank under the accession number CP066167. The proteomics data have been deposited to the Proteome Xchange Consortium via the PRIDE (55) partner repository with the data set identifier PXD028001. The strain *Spongiibacter nanhainus* CSC3.9 was deposited at the Korean Collection for Type Cultures (KCTC) with accession number KCTC 72889.

10.1128/msystems.01279-21.1 TEXT S1 Detailed description of the novel species *Spongiibacter nanhainus* sp. nov. Download Text S1, DOCX file, 0.01 MB. Copyright © 2022 Shan et al. 2022 Shan et al. https://creativecommons.org/licenses/by/4.0/ This content is distributed under the terms of the Creative Commons Attribution 4.0 International license.
