# Supplementary material for: A Deep-Sea Bacterium Senses Blue Light via a BLUF-Dependent Pathway
Source: mSystems. 2022 Feb 1;7(1):e01279-21. doi: 10.1128/msystems.01279-21 (PMC8805636; doi:10.1128/msystems.01279-21)
Supplement: FIG S1 [file msystems.01279-21-sf001.docx]

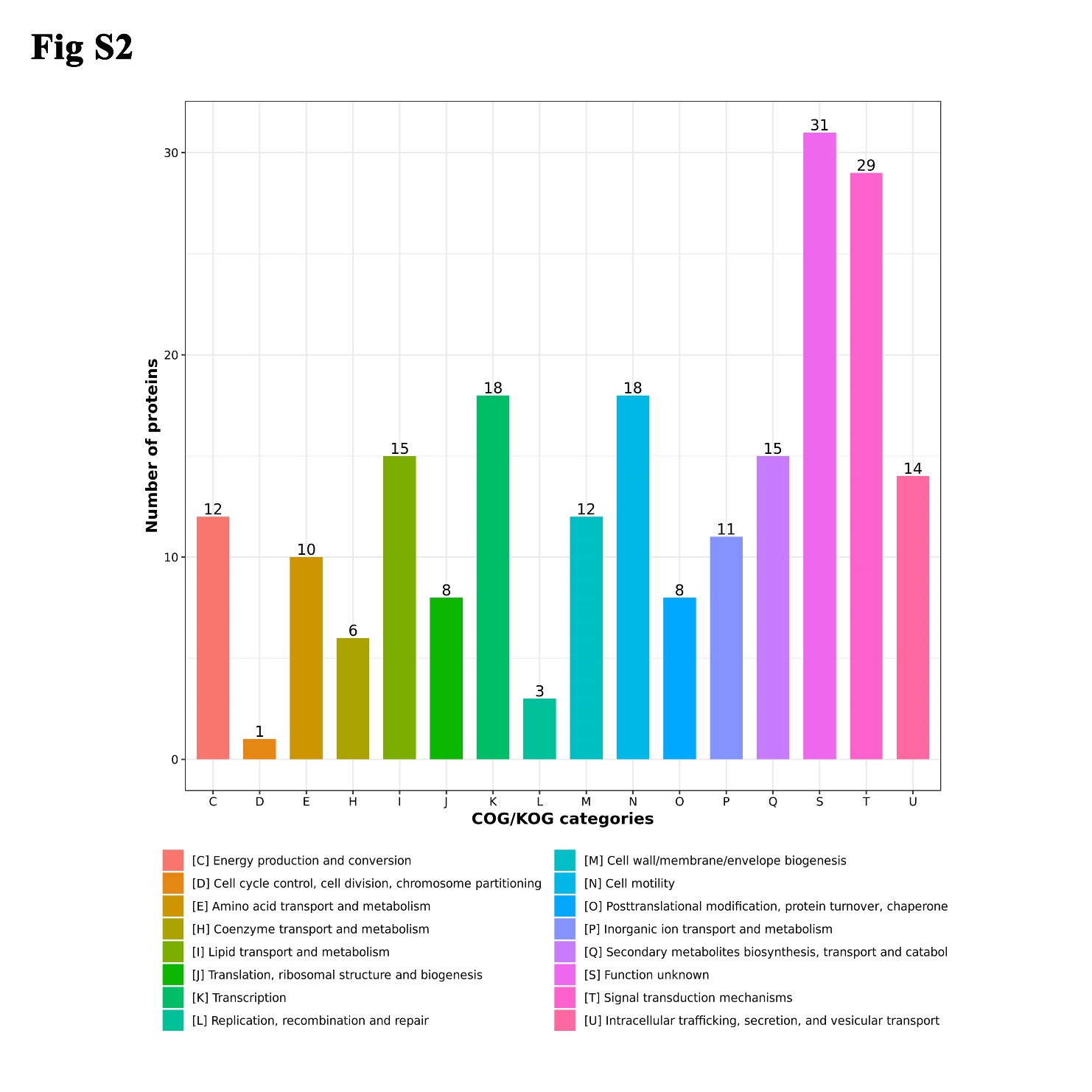


**FIG S1** COG analysis of proteomic differential proteins under the condition of blue light versus dark.
