# Supplementary material for: A Deep-Sea Bacterium Senses Blue Light via a BLUF-Dependent Pathway
Source: mSystems. 2022 Feb 1;7(1):e01279-21. doi: 10.1128/msystems.01279-21 (PMC8805636; doi:10.1128/msystems.01279-21)
Supplement: FIG S2 [file msystems.01279-21-sf002.docx]

**
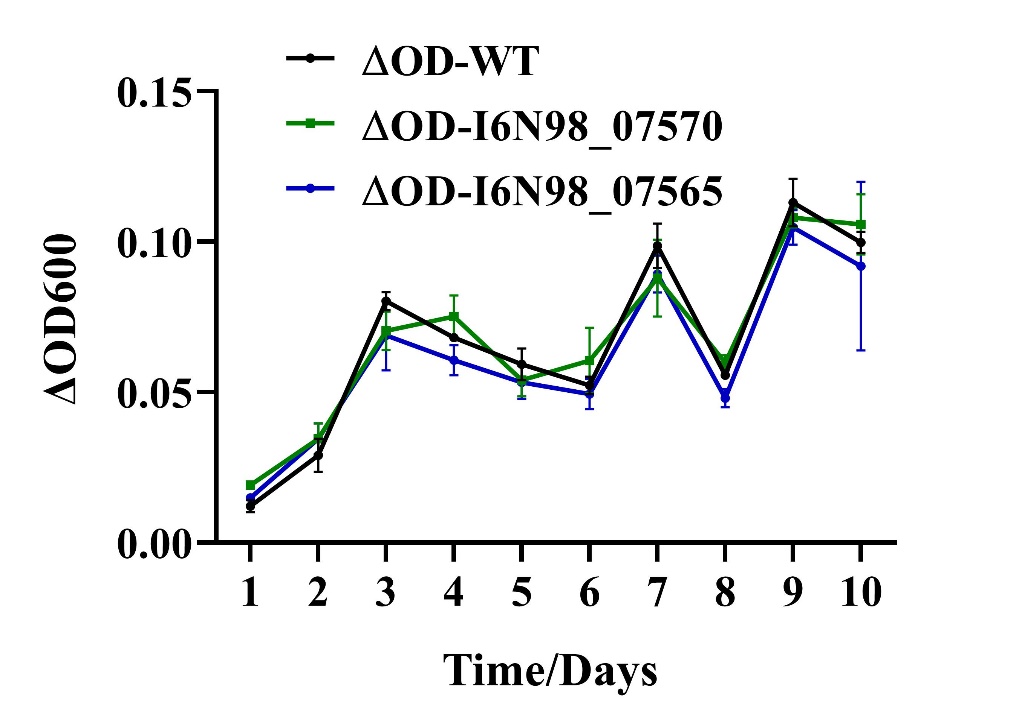
**

**FIG S2** Effects of blue light exposure on the growth of the wildtype (WT) and two bacteriophytochromes-like genes (I6N98_07570 and I6N98_07565) deletion mutant of strain CSC3.9 when respectively cultured under blue light and dark conditions. ΔOD600 indicates the differential OD_600_ value of cells (wildtype or deletion mutant) grown in the blue light and dark conditions.
