# Supplementary material for: A Deep-Sea Bacterium Senses Blue Light via a BLUF-Dependent Pathway
Source: mSystems. 2022 Feb 1;7(1):e01279-21. doi: 10.1128/msystems.01279-21 (PMC8805636; doi:10.1128/msystems.01279-21)
Supplement: FIG S3 [file msystems.01279-21-sf003.docx]

**
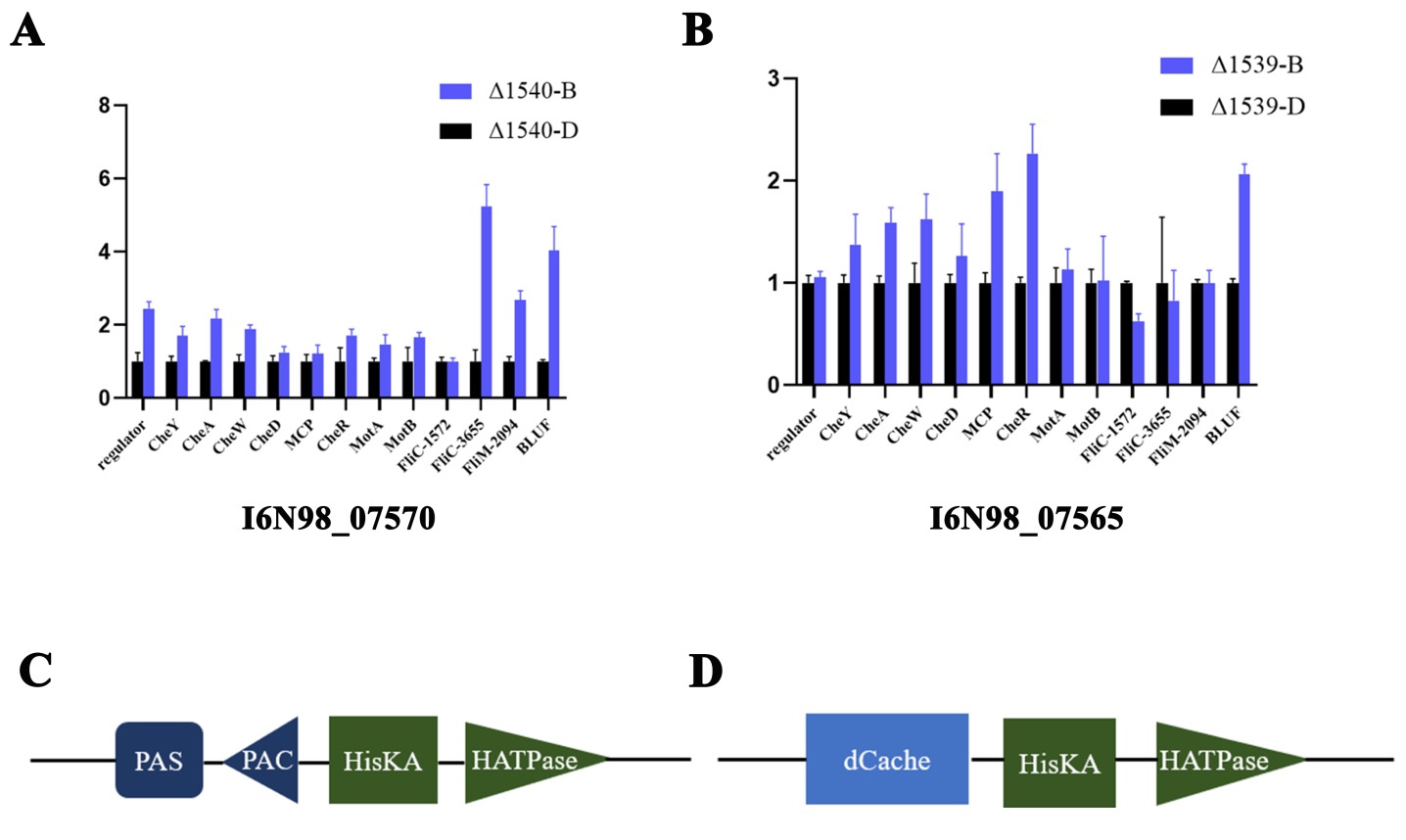
**

**FIG S3** Genetic analysis and structural prediction of two bacteriophytochrome-like proteins identified in strain CSC3.9 via proteomic assays. (A, B) qRT-PCR analysis of the expression of genes encoding proteins associated with chemotaxis in *07570-bacteriophytochrome* deletion mutant strain Δ*1540* (07570-bacteriophytochrome) and *07565-bacteriophytochrome* deletion mutant strainΔ*1539* (07565-bacteriophytochrome) under blue light (indicating with B) and dark (indicating with D) conditions. (C, D) Structural domain prediction of 07570-bacteriophytochrome and 07565-bacteriophytochrome based on SMART database.
