# Supplementary material for: A Deep-Sea Bacterium Senses Blue Light via a BLUF-Dependent Pathway
Source: mSystems. 2022 Feb 1;7(1):e01279-21. doi: 10.1128/msystems.01279-21 (PMC8805636; doi:10.1128/msystems.01279-21)
Supplement: FIG S4 [file msystems.01279-21-sf004.docx]

**
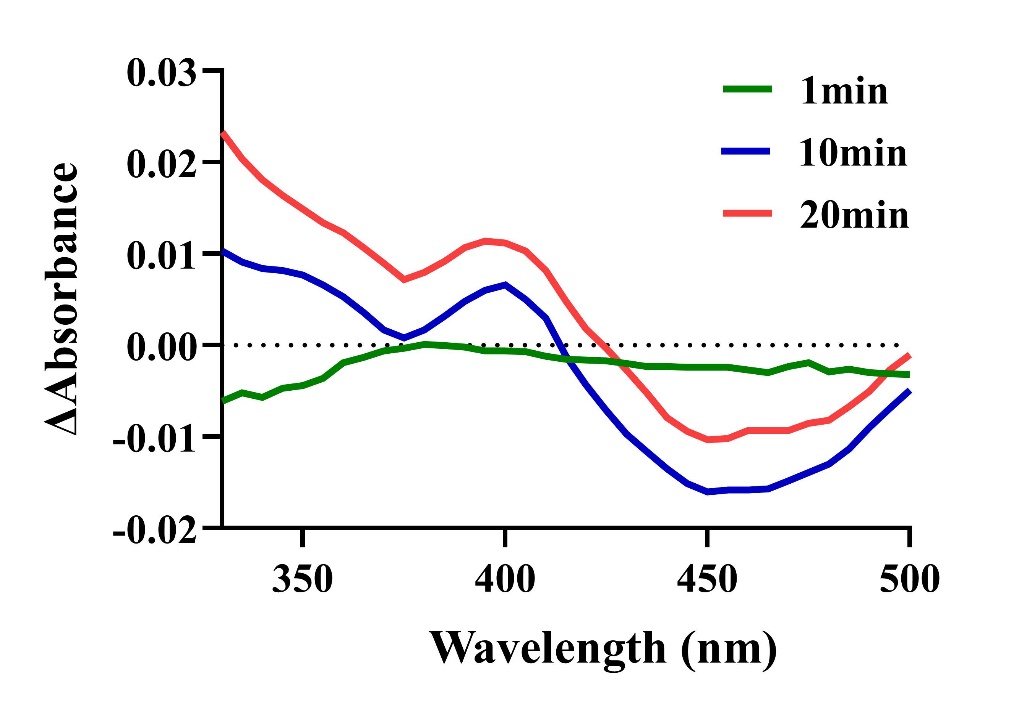
**

**FIG S4** The differential spectrum between treatments of BLUF with dark and irradiation for 1 min, 10 min or 20 min by white light at an intensity of 450 μ mol m^−2^ s^−1^. ΔAbsorbance in the Y-axis indicates the difference value of absorbance of BLUF under white light irradiation and dark condition.
