# Supplementary material for: A Deep-Sea Bacterium Senses Blue Light via a BLUF-Dependent Pathway
Source: mSystems. 2022 Feb 1;7(1):e01279-21. doi: 10.1128/msystems.01279-21 (PMC8805636; doi:10.1128/msystems.01279-21)
Supplement: TABLE S5 [file msystems.01279-21-st005.docx]

**Supplementary Table S5** The distribution of *BLUF* gene identified in different phyla of isolated marine microorganisms deposited in the IMG database

| **Phylum** | **Number of *BLUF* gene** |
| --- | --- |
| *Acidobacteria* | 10 |
| *Bacteroidetes* | 171 |
| *Proteobacteria* | 832 |
| *Verrucomicrobia* | 1 |
| *unclassified* | 1 |
| *Cyanobacteria* | 8 |
| *Lentisphaerae* | 1 |
| *Planctomycetes* | 6 |
| *Actinobacteria* | 10 |
| *Nitrospirae* | 2 |
| *Rhodothermaeota* | 6 |
| Total | 1048 |
