# Supplementary material for: A Deep-Sea Bacterium Senses Blue Light via a BLUF-Dependent Pathway
Source: mSystems. 2022 Feb 1;7(1):e01279-21. doi: 10.1128/msystems.01279-21 (PMC8805636; doi:10.1128/msystems.01279-21)
Supplement: TABLE S1 [file msystems.01279-21-st001.docx]

**Supplementary Table S1** Differential physiological and phenotypic characteristics of the strain CSC3.9 and the type strain HAL40b^T^ (+, Positive result or growth; -, negative result)

| Characteristic | *Spongiibacter nanhaius* CSC3.9^T^ | *Spongiibacter marinus* HAL40b^T^ |
| --- | --- | --- |
| Isolation source | cold seep sediment | Marine sponge |
| Cell size (width × length) (μm) | 0.4-0.6×0.8-2.3 | 0.4-0.6×1-2 |
| Motility | + | ＋ |
| Conditions for growth |  |  |
| Temperature [optimum] (°C) | 10-42[30-37]* | 10-40 [20-30] |
| pH [optimum] | 5-10[6-8] | 6.5-9.5 [7-9] |
| NaCl tolerance [optimum] (%) | 2-9[5] | 1-7 [3] |
| Hydrolysis of Tween 40 | － | ＋ |
| DNA G+C content (mol%) | 57.12 | 69.1 |
| Oxidase and catalase | ＋ | ＋ |
| Major fatty acids  (>10%) | C _17:1_ *ω*8*c* | C _17:1_ *ω*8*c* |
| Polar lipids | PE; DPG; PG | DPG; PG; APL |
| Utilization of  Acetate  Maltose  Fructose  D-mannose  D-Glucose  Pyruvate  Sucrose | +  -  +  -  +  +  + | +  -  -  -  -  -  - |

*****The number shown in [ ] indicates the optimum conditions for the growth of corresponding strain.
