# Supplementary material for: A Deep-Sea Bacterium Senses Blue Light via a BLUF-Dependent Pathway
Source: mSystems. 2022 Feb 1;7(1):e01279-21. doi: 10.1128/msystems.01279-21 (PMC8805636; doi:10.1128/msystems.01279-21)
Supplement: TABLE S2 [file msystems.01279-21-st002.docx]

**Supplementary Table S2** The main fatty acids (%) of the strain CSC3.9^T^ compared with its closest type stain HAL40b^T^. Strains: 1, CSC3.9^T^ (all data from this study); 2, *Spongiibacter marinus* HAL40b^T^ (all data from this study).

| **Fatty acid** | **Percentage (w/v) of total fatty acids**  **1 2** | |
| --- | --- | --- |
| Branched: |  |  |
| C**_11:0_** 3-OH | 1.68 | 7.77 |
| C**_9:0_** | 0.32 | 3.7 |
| C**_11:0_** | 4.71 | 1.49 |
| C**_18:1_** *ω7c*/C**_18:1_** *ω6c* | 9.97 | 6.46 |
