# Supplementary material for: A Deep-Sea Bacterium Senses Blue Light via a BLUF-Dependent Pathway
Source: mSystems. 2022 Feb 1;7(1):e01279-21. doi: 10.1128/msystems.01279-21 (PMC8805636; doi:10.1128/msystems.01279-21)
Supplement: TABLE S3 [file msystems.01279-21-st003.docx]

**Supplementary Table S3** Strains, plasmids and primers used for vector construction

| **Strains** | **Relevant genotype, description or sequence** | **Reference or source** |
| --- | --- | --- |
| *Spongiibacter nanhainus CSC3.9* | Wild type, isolated from deep sea cold seep water | this work |
| *S. nanhainus CSC3.9 ΔBLUF* | Deletion of *BLUF* (I6N98_15960) in strain CSC3.9 | this work |
| *S. nanhainus CSC3.9 Δ1540* | Deletion of *07570-bacteriophytochrome* (I6N98_07570) in strain CSC3.9 | this work |
| *S. nanhainus CSC3.9 Δ1540* | Deletion of *07565-bacteriophytochrome* (I6N98_07565) in strain CSC3.9 | this work |
| *Escherichia coli* DH5α | hsdR recA lacZYA ϕ80 lacZΔM15 | this work |
| *Escherichia coli* SY327 | F^−^ araD *Δ(lac-proAB)* argE(Am) rif nalA recA56 | (1) |
| *Escherichia coli* S17-1 | thi pro hsdr - hsdM + recA [ chr:: RP4-2-Tc::Mu-Km::Tn7] | (2) |
| **Plasmids** |  |  |
| pEX18Gm | 5.8 kb plasmid, Gm^R^, suicide vector | (3) |
| pEX-18Gm-KO-*1539* | Gm^R^, an overlap of 2025 bp inserted into pEX18Gm using *Kpn*Ⅰand *Bam*HⅠ | this work |
| pEX-18Gm-KO-*1540* | Gm^R^, an overlap of 1187 bp inserted into pEX18Gm using *Hin*d Ⅲand *Bam*HⅠ | this work |
| pEX-18Gm-KO-*BLUF* | Gm^R^, an overlap of 428 bp inserted into pEX18Gm | this work |
| pET28a | 5.6 kb plasmid, Kan^R^, expression vector | this work |
| pET28a-BLUF | *BLUF* gene cloned into pET28a | this work |
| **Primers (5’-3’)** |  |  |
| 1539up-F | CGCGGATCCGCGCGCGCGTTCCAAAGATAGTG |  |
| 1539up-R | GGGCCAACTGAGTTCCTACCTGACGACGCAGCCACTAACT |  |
| 1539down-F | AGTTAGTGGCTGCGTCGTCAGGTAGGAACTCAGTTGGCCC |  |
| 1539down-R | CGGGGTACCCCGGGAAGATCTCGAGCTTGCCA |  |
| 1540up-F | CGCGGATCCGCGTCGGGTCCGGATGAAAAAGG |  |
| 1540up-R | GTCAGTACGGAAAGCCTGCTGCTAGAATCTATATACATAC |  |
| 1540down-F | GTATGTATATAGATTCTAGCAGCAGGCTTTCCGTACTGAC |  |
| 1540down-R | CCCAAGCTTGGGTCAAGGTCAATACCCACGCC |  |
| BLUFup-F | GAGCTCGGTACCCGGGGATCCTGCTTCGTCAGTACTGGCAGC |  |
| BLUFup-R | ACGTATTGGAGGGACCACTTAAGAGTTAGAACTATCTCTG |  |
| BLUFdown-F | CAGAGATAGTTCTAACTCTTAAGTGGTCCCTCCAATACGT |  |
| BLUFdown-R | CAGGTCGACTCTAGAGGATCCATTGAGTACAATCTCTACAACCAGCAT |  |
| 28aBLUF-F | CAGCAAATGGGTCGCGGATCCATGTACCTAGCGCGTCTAATATACACC |  |
| 28aBLUF-R | ACGGAGCTCGAATTCGGATCCCTACACGGCTGGCAGCACTT |  |

**References related to this table**

1. Goldberg I, Mekalanos JJ. 1986. Cloning of the *Vibrio cholerae recA g*ene and construction of a *Vibrio cholerae recA* mutant. J Bacteriol 165:715-722. doi:DOI 10.1128/jb.165.3.715-722.1986.

2. Schafer A, Tauch A, Jager W, Kalinowski J, Thierbach G, Puhler A. 1994. Small mobilizable multi-purpose cloning vectors derived from the *Escherichia coli* plasmids pK18 and pK19: selection of defined deletions in the chromosome of *Corynebacterium glutamicum*. Gene 145:69-73. doi:10.1016/0378-1119(94)90324-7.

3. Hoang TT, Karkhoff-Schweizer RR, Kutchma AJ, Schweizer HP. 1998. A broad-host-range Flp-FRT recombination system for site-specific excision of chromosomally-located DNA sequences: application for isolation of unmarked *Pseudomonas aeruginosa* mutants. Gene 212:77-86. doi:Doi 10.1016/S0378-1119(98)00130-9.
