# Supplementary material for: A Deep-Sea Bacterium Senses Blue Light via a BLUF-Dependent Pathway
Source: mSystems. 2022 Feb 1;7(1):e01279-21. doi: 10.1128/msystems.01279-21 (PMC8805636; doi:10.1128/msystems.01279-21)
Supplement: TABLE S4 [file msystems.01279-21-st004.docx]

**Supplementary Table S4** Primers used for qRT-PCR

| **Primer name** | **Nucleotide Sequence (5’-3’)** |
| --- | --- |
| 16S-F | TTTAACCTTGCGGCCGTACT |
| 16s-R | AGGAACATCAGTGGCGAAGG |
| 960-F | GCACAACAACCGTGAGCATT |
| 960-R | CGGTCTCGGGAAGATAAGCC |
| 850-F | CGGCAACTGGTTTCCATGAC |
| 850-R | ATCGGGGTGAAGCGAAACTT |
| 845-F | TGATGTTCAGGCCCGTATCG |
| 845-R | GTTCGGCGGAGACGGATAAT |
| 840-F | GGACGCAGTTTCCGAGGTAT |
| 840-R | CGTTTGCAGCGACGATTTCA |
| 825-F | GGGGCGGTAAGATAATGCGA |
| 825-R | AATACTGGTGTCGTGCAGGG |
| 805-F | TCAGCGCGATAACCACCAAT |
| 805-R | CCAGTTTCTGCACTCCGGTA |
| 800-F | ACCGCAGTTCGTGCTAAAGA |
| 800-R | CAACAAGGTTGTCGGCACTG |
| 650-F | CTTTCGCTCCAACTGGGACT |
| 650-R | GTGACCTCAACCCGACGATT |
| 645-F | GATCGGCCTGGTCCAAATGA |
| 645-R | GTTGACCGCCTCCAGAATCA |
| 130-F | TGGTGTCGGCGATTAACCAA |
| 130-R | TTGGTCTGTGATGTACCGGC |
