# Supplementary material for: A Deep-Sea Bacterium Senses Blue Light via a BLUF-Dependent Pathway
Source: mSystems. 2022 Feb 1;7(1):e01279-21. doi: 10.1128/msystems.01279-21 (PMC8805636; doi:10.1128/msystems.01279-21)
Supplement: TEXT S1 [file msystems.01279-21-t0001.docx]

**Description of *Spongiibacter nanhainus* sp. nov.** *Spongiibacter* *nanhainus* (nan.hai'us. N.L. masc. adj. nanhainus pertaining to Nanhai, where the type strain was isolated).

Based on the 16S rRNA gene, phylogenetic analysis with three tree-making algorithms showed that strain CSC3.9 belonged to the class Gammaproteobacteria. Strain CSC3.9 was most closely related to *Spongiibacter marinus* strain HAL40b^T^ (95.75% similarity). The phylogenetic tree completed by three different algorithms revealed that *Spongiibacter nanhainus* CSC3.9 formed a fairly robust clade (bootstrap value 91%) with type strains of *Spongiibacter marinus*, *Spongiibacter tropicus* and *Spongiibacter taiwanensis*. The clade could be further divided into two subclades: one of which consisted of strain *Spongiibacter nanhainus* CSC3.9 and *Spongiibacter marinus* strain HAL40b^T^ with 76% bootstrap support. The 16S rRNA gene-based sequence similarity and phylogenetic tree analysis supported the assignment of strain CSC3.9 to a novel species of the genus *Spongiibacter*.

In addition to the description of the blue light response characteristic, the physiological and genome properties of strain CSC3.9 were determined as following (Supplementary Tables S1 and S2). Cells were facultative anaerobic, Gram-reaction-negative, rod-shaped approximately 0.8-2.3 µm long and 0.4-0.6 µm wide with single polar flagellum. Growth detected at temperatures between 10 °C and 42 °C, but not at 10 °C or below, nor at 42 °C or above. The optimum salinity of strain CSC3.9 was 4% and the optimum pH was 6.8. Positive for oxidase and catalase. Growth was stimulated by the supplement of acetate, fructose, D-Glucose, pyruvate or sucrose. Major fatty acids and the main quinone of strain CSC3.9 were C_17:1_ *ω*8*c* and ubiquinone 8 (Q-8). Genome size was 4.08 Mbp, with 3,730 predicted genes, and a G+C content of 56.5%. Strain CSC3.9 had an ANIb of 71.05% with *Spongiibacter marinus* and an AAI of 71% with *Zhongshania aliphaticivorans*. *Spongiibacter nanhainus* CSC3.9 was deposited at Korean Collection for Type Cultures (KCTC) with accession number KCTC 72889. The type strain, *Spongiibacter nanhainus*, was isolated from a cold seep (at depth of 1,121m) in the South China Sea.
